# Large Pleuritic Effusions in Phthisis

**Published:** 1875-12

**Authors:** 


Large Pleuritic Effusions in Phthisis.—M. Leudet (*Gaz. Hebdom.*, September 3, 1875) read at the meeting of the French Science Association at Nantes a paper upon this subject, which he terminated with the following conclusions: 1. In the course of pulmonary tuberculosis the pleura may become filled with an effusion. 2. While this may be serous, purulent, or hemorrhagic, it is most commonly pseudo-membranous. 3. Pleurisy occupying the entire pleura is more often tubercular in its nature than idiopathic. 4. Patients dying during the existence of these effusions frequently present caverns and tubercles in part arrested, or cretaceous—*i. e.*, the lesions of regressive tuberculosis especially appertaining to irregular phthisis. More rarely the tuberculosis is double, and softened; and, more rarely still, only miliary tubercles may be met with. 5. The tnberculosis is not more extensive and advanced on the side of the effusion; it is even often less so. 6. The pleurisy does not generally induce death by its abundance. 7. Some patients die in a cachectic condition before the complete absorption of the effusion. 8. Two-thirds of these patients are cured of the effusion. 9. The cure is in general slower than in the non-tuberculosis. 10. Purulent pleuritic tubercular patients are susceptible of cure. 11. Abundant effusion does not, for the most part, increase pulmonary tuberculosis, and in general it does not induce a more rapid evolution of tuberculosis in the lung of the side of the effusion than in that of the opposite side. 12. Purulent pleurisy does not seem to accelerate the development of tuberculosis.—*Medical Times and Gazette*, Sept. 18, 1875.

